# Superior Clinical and Economic Value of IL‐23 Inhibitors Versus Adalimumab Biosimilars in Plaque Psoriasis

**DOI:** 10.1111/ijd.70371

**Published:** 2026-03-29

**Authors:** Gennaro Marco Falco, Elena Ippoliti, Niccolò Gori, Giacomo Caldarola, Clara De Simone, Nicoletta Di Giorgi, Paulo Gabriel Longo, Marco Galluzzo, Luca Bianchi, Lorenzo Marcelli, Marina Talamonti, Maria Vittoria Cannizzaro, Lucia Di Nardo, Ketty Peris

**Affiliations:** ^1^ Dermatologia, Dipartimento Universitario di Medicina e Chirurgia Traslazionale Università Cattolica del Sacro Cuore Rome Italy; ^2^ UOC Dermatologia, Dipartimento di Scienze Mediche e Chirurgiche Fondazione Policlinico Universitario A. Gemelli IRCCS Rome Italy; ^3^ Real World Data Facility, Gemelli Generator Fondazione Policlinico Universitario Agostino Gemelli IRCCS Rome Italy; ^4^ Department of Systems Medicine University of Rome Tor Vergata Rome Italy; ^5^ Dermatology Unit Fondazione Policlinico Tor Vergata Rome Italy; ^6^ Immunology Research Core Facility, Gemelli Science and Technology Park (GSTeP) Fondazione Policlinico Universitario A. Gemelli‐IRCCS Rome Italy

**Keywords:** adalimumab, anti‐IL‐23, NNT, PASI90, psoriasis

## Abstract

**Background:**

In recent years, the introduction of interleukin (IL)‐23 inhibitors has revolutionized psoriasis treatment, allowing the majority of patients to achieve a long‐term, complete clinical control of the disease. Nevertheless, there are no real‐world studies exploring the cost benefit ratio of these new drugs compared to the currently most prescribed therapies as adalimumab biosimilars.

**Methods:**

We performed a retrospective study to evaluate the clinical effectiveness, measured as Psoriasis Area and Severity Index (PASI)90‐number needed to treat (NNT) and the cost‐effectiveness of IL‐23 inhibitors (guselkumab, risankizumab, tildrakizumab) in patients with moderate to severe psoriasis as compared to adalimumab biosimilars (imraldi, hyrimoz, yuflima, and amgevita). Assessments were performed at Weeks 16 and 52.

**Results:**

Our analysis included 616 patients with moderate to severe psoriasis who had been treated with adalimumab biosimilars or an anti‐IL‐23 inhibitor for at least 16 weeks. Overall, IL‐23 inhibitors were shown to be more effective compared to adalimumab biosimilars, with significantly lower NNT. Risankizumab achieved the highest PASI90 response rates, whereas guselkumab had the most favorable incremental cost per PASI90 responder at Weeks 16 and 52.

**Conclusions:**

Our study highlights the substantial clinical benefit of IL‐23 inhibitors over adalimumab biosimilars, with guselkumab as the more cost‐effective option than the other IL‐23 inhibitors for the real‐world management of moderate‐to‐severe psoriasis.

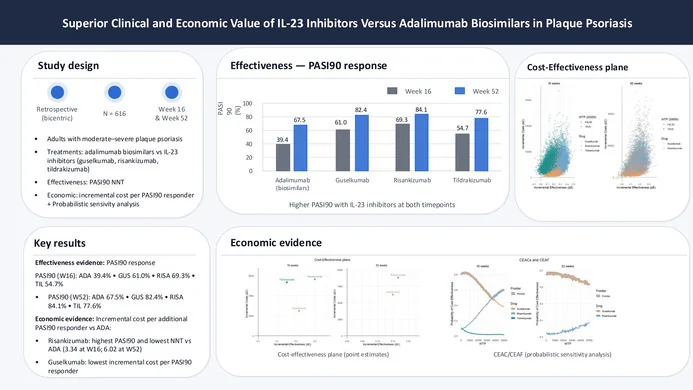

## Graphical abstract

A bicentric retrospective real‐world study (*N* = 616) comparing IL‐23 inhibitors (guselkumab, risankizumab, tildrakizumab) with adalimumab biosimilars in moderate‐to‐severe plaque psoriasis. It presents PASI90 response at Weeks 16 and 52, NNT, and cost‐effectiveness (incremental cost per responder, CE plane, CEAC/CEAF).

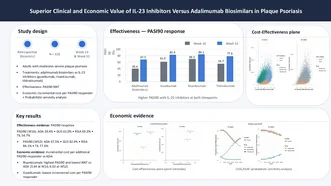

## 1 Introduction

Psoriasis is a chronic inflammatory skin disorder with a prevalence of 1%–3% worldwide, strongly associated with multiple comorbidities that contribute to its great impact on quality of life (QoL) [1].

In recent years, increased understanding of its pathogenesis has revolutionized the therapeutic landscape of psoriasis, leading to the development of novel biological therapies that often achieve complete skin clearance and improvement in QoL for most patients [2]. Recent studies have focused on the role of interleukin (IL)‐23 in the pathogenesis of psoriasis. IL‐23, a member of the IL‐12 cytokine family, plays a key role in many immune‐mediated diseases, including psoriasis and psoriatic arthritis, by sustaining a pro‐inflammatory environment through modulation of immune cells. IL‐23 is produced by tissue resident myeloid cells, promoting the expansion of T helper (Th)‐17 lymphocytes, with subsequent production of IL‐17A and other inflammatory cytokines [3].

Humanized monoclonal antibodies targeting the p19 subunit of IL‐23—guselkumab, tildrakizumab, and risankizumab—are approved for the treatment of moderate‐to‐severe psoriasis, with regulatory approval granted by the United States Food and Drug Administration (FDA) and the European Medicines Agency (EMA) in 2017, 2018 and in 2019, respectively [4]. In pivotal phase III clinical trials, risankizumab demonstrated Psoriasis Area and Severity Index (PASI) 90 response rates of 75.3% and 74.8% at Week 16 in UltIMMa‐1 and UltIMMa‐2, respectively. Guselkumab achieved PASI90 response in 73.3% and 70.0% in VOYAGE 1 and 2; while tildrakizumab reached 36% and 39% in reSURFACE 1 and 2, respectively (*p* < 0.001). However, real‐world evidence remains limited [5]. Anti‐TNF agents represented the first approved class of biologics, and their biosimilars have been introduced into the market as a less expensive option compared to the recently developed IL‐23 and IL‐17 inhibitors [6]. Outcomes of anti‐TNF were lower compared to IL‐23 inhibitors in both randomized clinical trials and real‐world studies when considering the current standards of care, that is PASI90 response obtained at Week 16 and sustained in the long‐term [5, 7]. Indeed, achievement of PASI90 as well as high rates of drug survival are more frequently observed with the use of IL‐23 inhibitors than with anti‐TNF [8].

Direct or indirect comparisons between these two classes of agents are in favor of the IL‐23 inhibitors, though no data about the number‐needed‐to‐treat (NNT), with a direct comparison between these classes of agents have been reported so far. In addition, there is a lack of a direct comparative analysis linking the economic impact of anti IL‐23 drugs to their clinical benefit, an issue of considerable importance given the substantial economic burden these therapies impose on healthcare expenditure and the need for resource allocation [9].

We conducted a retrospective study including patients with moderate to severe psoriasis treated with IL‐23 inhibitors (guselkumab, risankizumab, and tildrakizumab) or adalimumab biosimilars (imraldi, hyrimoz, yuflima, and amgevita) to estimate the PASI90 NNT of IL‐23 inhibitors compared with adalimumab biosimilars, currently used as first‐line biologic therapy in most patients because of a high effectiveness/price ratio. In addition, the cost per PASI90 responder with guselkumab, risankizumab, and tildrakizumab for the treatment of moderate to severe psoriasis was evaluated and compared with that of adalimumab biosimilars.

## 2 Materials and Methods

This bicentric observational study was conducted at two University Hospitals: Fondazione Policlinico Universitario Agostino Gemelli IRCCS and Policlinico Universitario Tor Vergata in Rome.

Data on effectiveness were retrieved from patients aged ≥ 18 years, with moderate to severe psoriasis, treated with anti‐IL‐23 agents (guselkumab, risankizumab, and tildrakizumab) or adalimumab biosimilars for at least 16 weeks.

Treatment response was assessed as the percentage reduction in PASI score from baseline. A ≥ 90% improvement in PASI score from baseline (PASI90) was used as a criterion for treatment success, evaluated in the short‐ (week 16) and long‐term (week 52). For each treatment, the NNT to achieve one additional PASI90 response compared with adalimumab biosimilars was calculated as the reciprocal of the difference in estimated PASI90 response rates between each IL‐23 inhibitor (guselkumab, risankizumab, and tildrakizumab) and adalimumab biosimilars [10, 11].

In addition, using adalimumab biosimilars as reference, we calculated NNT for each inhibitor (guselkumab, risankizumab, and tildrakizumab) and the control group (adalimumab) in order to compare NNT scoring among IL‐23 inhibitors. The lower the NNT, the greater the effectiveness of the intervention compared to the selected comparator.

Effectiveness outcomes were analyzed using an “as observed” method at each time point. For Week 16 and Week 52, denominators included only patients with an available PASI assessment at the corresponding visit.

### 2.1 Cost Data

Drug consumption was evaluated by applying the ex‐factory prices of each product [12] net of temporary statutory discounts but excluding any confidential or region‐specific tender discounts (Table S1). The weighted average price (WAP) for adalimumab biosimilars was calculated based on the prescription frequency of the drugs imraldi, hyrimoz, yuflima (net ex‐factory price per unit: €308.98) and amgevita (net ex‐factory price per unit: €348.14) during the years 2020–2024, resulting in a WAP of €309.03. The administration regimens for the selected drugs, including the route of administration and the number of doses over the initial 16‐week treatment period, are illustrated in Table S1.

At the time of enrolment, patients reporting comorbidities, current medical conditions, and medical history of cancer were included in the study. The study was conducted in accordance with the Helsinki Declaration of 1975, as revised in 2013, and received the approval of the relevant Ethical Committees. The protocol was reviewed and approved by the Local Ethics Committee, Prot N.5601/23. All patients signed an informed consent.

### 2.2 Cost Analysis

The comparison between biological treatments was conducted using the estimated incremental cost per PASI90 responder, considering the difference in PASI90 response between each biologic and adalimumab biosimilars as the incremental effectiveness [13]. This analysis was performed at Weeks 16 and 52, but only for those biological treatments whose difference in effectiveness compared to adalimumab was statistically significant at the 95% confidence level. Incremental costs per PASI90 responder were estimated only for treatments whose incremental effectiveness versus adalimumab was statistically significant at the 95% confidence level. This was done to avoid unstable incremental cost per PASI90 responder estimates when incremental effectiveness is not statistically different from zero.

Following the point estimates comparison between biological treatments, a one‐way deterministic analysis and a probabilistic sensitivity analysis (PSA) were conducted to assess the robustness of the results. The one‐way deterministic sensitivity analysis focused on the impact of plausible variation in effectiveness and cost parameters on the incremental cost per PASI90 responder. Incremental effectiveness was varied using the lower and upper bounds of the 95% confidence intervals of the probability of achieving PASI90 for each biological treatment, while drug acquisition costs were varied by ±30% relative to base‐case estimates. PSA was conducted using 10,000 samples, assuming a normal distribution for incremental effectiveness, with point estimates of the absolute risk reduction as the means and standard deviations derived from the 95% confidence intervals for each comparison. A log‐normal distribution was assumed for incremental costs, with the mean set to the average cost difference between each treatment and adalimumab, and a standard deviation corresponding to a 30% variation in the cost of each biological treatment. A correlation of 0.4 was specified between incremental effectiveness and incremental cost for each biological treatment. Alternative combinations of cost variation percentages and correlation values were explored to account for additional uncertainty of these two parameters (Figures S1 and S2). A probabilistic sensitivity analysis was conducted, in which costs and effectiveness for each treatment, including adalimumab, were simulated using log‐normal and beta distributions, respectively. This analysis aimed to compare the probability of each treatment being cost‐effective, including the comparator in the analysis (Figure S3).

## 3 Results

We retrieved 616 patients with moderate to severe psoriasis, including 310 (50.3%) females and 306 (49.7%) males, with a mean age of 51.7 years (standard deviation [SD] ±15.2). Comorbidities comprised cardiovascular disease (175/616, 28.4%), obesity (121/616, 19.6%), and psoriatic arthritis (84/616, 13.6%). A family history of psoriasis was reported by 257/616 (41.7%) patients (Table S2).

Out of 616 patients, 200 (32.5%) were treated with guselkumab, 176 (28.6%) with risankizumab, 165 (26.8%) with adalimumab biosimilars and 75 with tildrakizumab (12.1%). Overall, 352 (57.1%) patients were naïve to biological therapies: 159 (45.2%) received adalimumab, 75 (21.3%) guselkumab, 52 (14.7%) tildrakizumab, and 66 (18.8%) risankizumab. Previous treatment with at least one biological therapy was detected in 264/616 (42.8%) patients.

Higher PASI90 response rates were observed in patients treated with IL‐23 inhibitors compared with those receiving adalimumab biosimilars. In the guselkumab group, PASI90 response was achieved by 61.0% (122/200) and 82.4% (159/193) of patients at Week 16 and 52, respectively. Similarly, risankizumab‐treated patients achieved PASI90 response in 69.3% (122/176) and 84.1% (122/145) of cases at Week 16 and 52, respectively. In the tildrakizumab cohort, PASI90 was observed in 54.7% (41/75) and 77.6% (45/58) of patients at Week 16 and 52, respectively. In the adalimumab biosimilars group, 39.4% (65/165) and 67.5% (56/83) of patients achieved PASI90 response at Week 16 and week 52, respectively (Table S2).

Risankizumab demonstrated the lowest NNT to achieve PASI90 at Week 16 (NNT = 3.34, 95% CI = 2.50–5.04), a finding that was maintained over the long‐term follow‐up period (Week 52) (NNT = 6.00; 95% CI = 3.62–17.49). When comparing anti IL‐23 agents collectively (tildrakizumab, risankizumab, and guselkumab) with adalimumab at each time point, the lowest NNT (indicating the highest efficacy) was observed at Week 16 (NNT = 4.202, 95% CI = 3.08–6.59, *p* < 0.0001) (Table 1).

**TABLE 1:** PASI90 response rates and PASI90 NNT as calculated at Weeks 16 and 52.

Treatment	PASI 90 response rate (%[*n*])	PASI90 NNT (95% CI)	RR (95% CI)	*p*	PASI90 response rate (%[*n*])	PASI90 NNT (95% CI)	RR (95% CI)	*p*
	Week 16				Week 52			
Adalimumab	39.4 (65/165)	Ref.	Ref.	Ref.	67.5 (56/83)	Ref.	Ref.	Ref.
Tildrakizumab	54.7 (41/75)	6.54 (3.49 benefit to 53.86 benefit)	0.75 (0.57–0.98)	**0.0402**	77.6 (45/58)	9.9 (20.36 harm to ∞ to 3.98 benefit)	0.69 (0.39–1.22)	0.201
Guselkumab	61.0 (122/200)	4.63 (3.16 benefit to 8.66 benefit)	0.64 (0.52–0.79)	**< 0.0001**	82.4 (159/193)	6.71 (3.93 benefit to 22.81)	0.54 (0.35–0.84)	**0.0057**
Risankizumab	69.3 (122/176)	3.34 (2.50 benefit to 5.04 benefit)	0.51 (0.39–0.65)	**< 0.0001**	84.1 (122/145)	6.02 (3.62 benefit to 17.49 benefit)	0.48 (0.29–0.79)	**0.0038**

*Note:* Bold values indicate statistical significance at *p* < 0.05.

### 3.1 Cost Analysis—Incremental Cost Per PASI90 Responder

Risankizumab had the highest average cost at both Week 16 and 52, corresponding to €8745.24 and €17,490.48, respectively, based on its dosing schedule, and the highest incremental effectiveness compared to adalimumab biosimilars (Table 2). At Week 52, the effectiveness of tildrakizumab was not significantly different from that of adalimumab, so it was excluded from analysis.

**TABLE 2:** Incremental cost, incremental effectiveness, number needed to treat (NNT), and incremental cost per PASI90 responder for treatments included in the analysis at 16 weeks (a) and 52 weeks (b).

Drug	Cost	Incremental cost	Incremental effectiveness	NNT	Incremental cost per PASI90 responder
**(a)**	**16 weeks**				
Adalimumab	€3090.30				
Guselkumab	€5585.52	€2495.22	0.216	4.63	€11,552.87
Risankizumab	€8745.24	€5654.94	0.299	3.34	€18,887.50
Tildrakizumab	€8443.20	€5352.90	0.153	6.55	€35,061.50
**(b)**	**52 weeks**				
Adalimumab	€8652.84				
Guselkumab	€14,894.72	€6241.88	0.149	6.70	€41,820.60
Risankizumab	€17,490.48	€8837.64	0.167	6.00	€53,025.84

As shown in the cost‐effectiveness plane (Figure 1), the data points are all located in the North East quadrant, where the *x*‐axis represents incremental effectiveness (PASI90 responders gained) and the *y*‐axis represents incremental cost. This means that every biological treatment was more effective than adalimumab but at a higher cost in both time intervals. Moreover, at Week 16, guselkumab dominated tildrakizumab, since guselkumab had a lower incremental cost compared to adalimumab than tildrakizumab, while simultaneously demonstrating higher incremental effectiveness.

**FIGURE 1:**
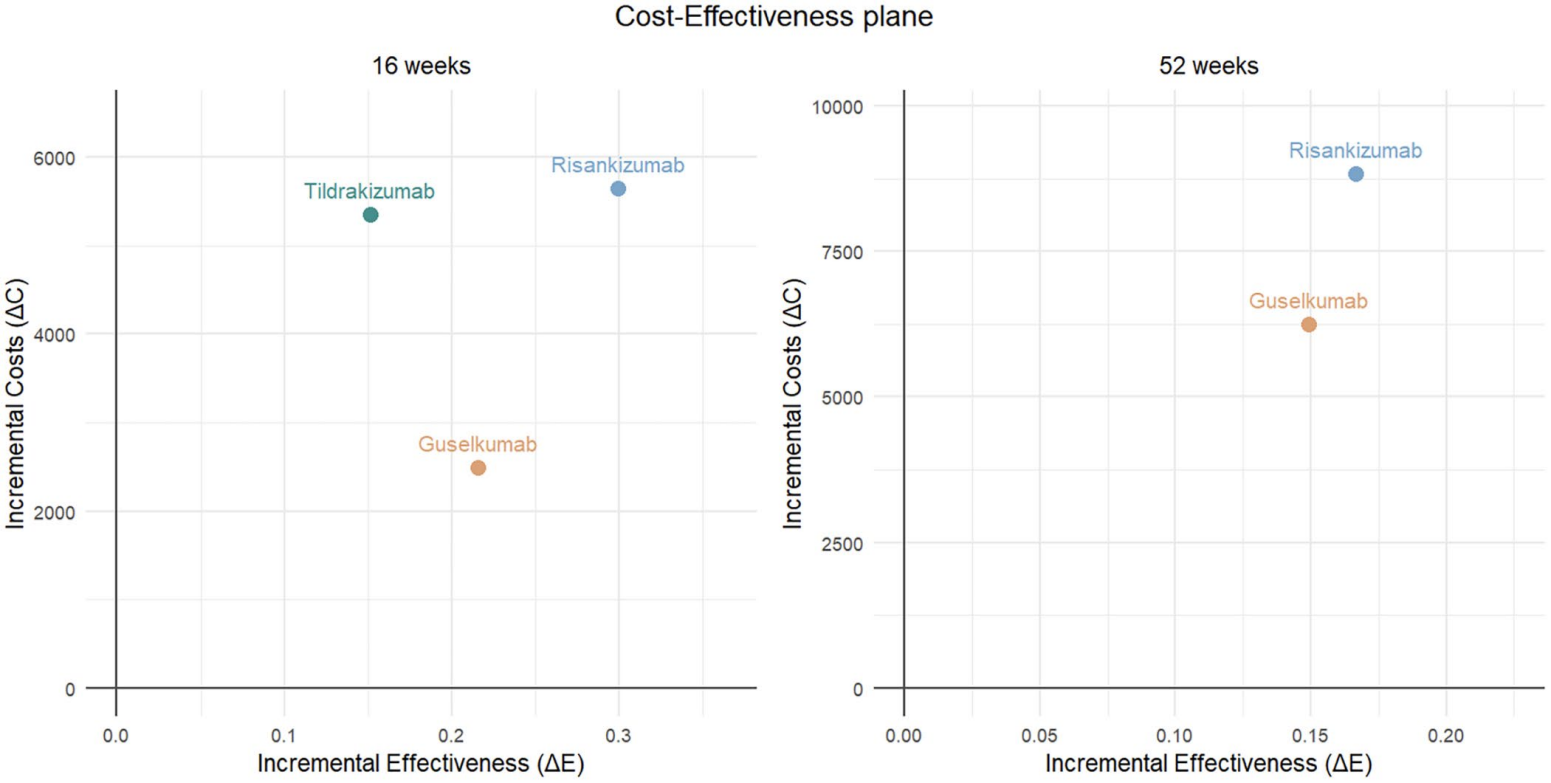
Cost‐effectiveness plane showing the incremental cost (*y*‐axis) versus incremental effectiveness (*x*‐axis) for the treatments compared to the reference (adalimumab biosimilars) at 16 and 52 weeks. Each point represents a treatment option (guselkumab, risankizumab, and tildrakizumab). All treatments are compared against adalimumab as the reference comparator.

At Week 16, guselkumab showed the lowest incremental cost per PASI90 responder gained (€11,552.87), followed by risankizumab (€18,887.50) and tildrakizumab. At 52 weeks, guselkumab again showed the lowest incremental cost per PASI90 responder, with a value of €41,820.60. Mathematically, these incremental costs per PASI90 responder correspond to the slope of the line connecting adalimumab (set at the origin of the cost‐effectiveness plane) to each alternative biological treatment.

### 3.2 Sensitivity Analyses

The results of the one‐way deterministic sensitivity analysis are shown in the tornado diagram (Figure 2). Horizontal bars show the variation in incremental cost per PASI90 responder with variation in the value of the parameter. At 16 weeks, drug acquisition cost was the main driver of the incremental cost per PASI90 responder for guselkumab and risankizumab, whereas for tildrakizumab, the primary driver was the probability of achieving PASI90. Across comparisons with adalimumab, guselkumab was associated with a relatively lower cost per responder compared with the other biologics. Similar patterns were observed at 52 weeks, with drug costs remaining the main driver for guselkumab and risankizumab. Across comparisons with adalimumab at both time points, guselkumab was associated with a relatively lower cost per responder compared with risankizumab.

**FIGURE 2:**
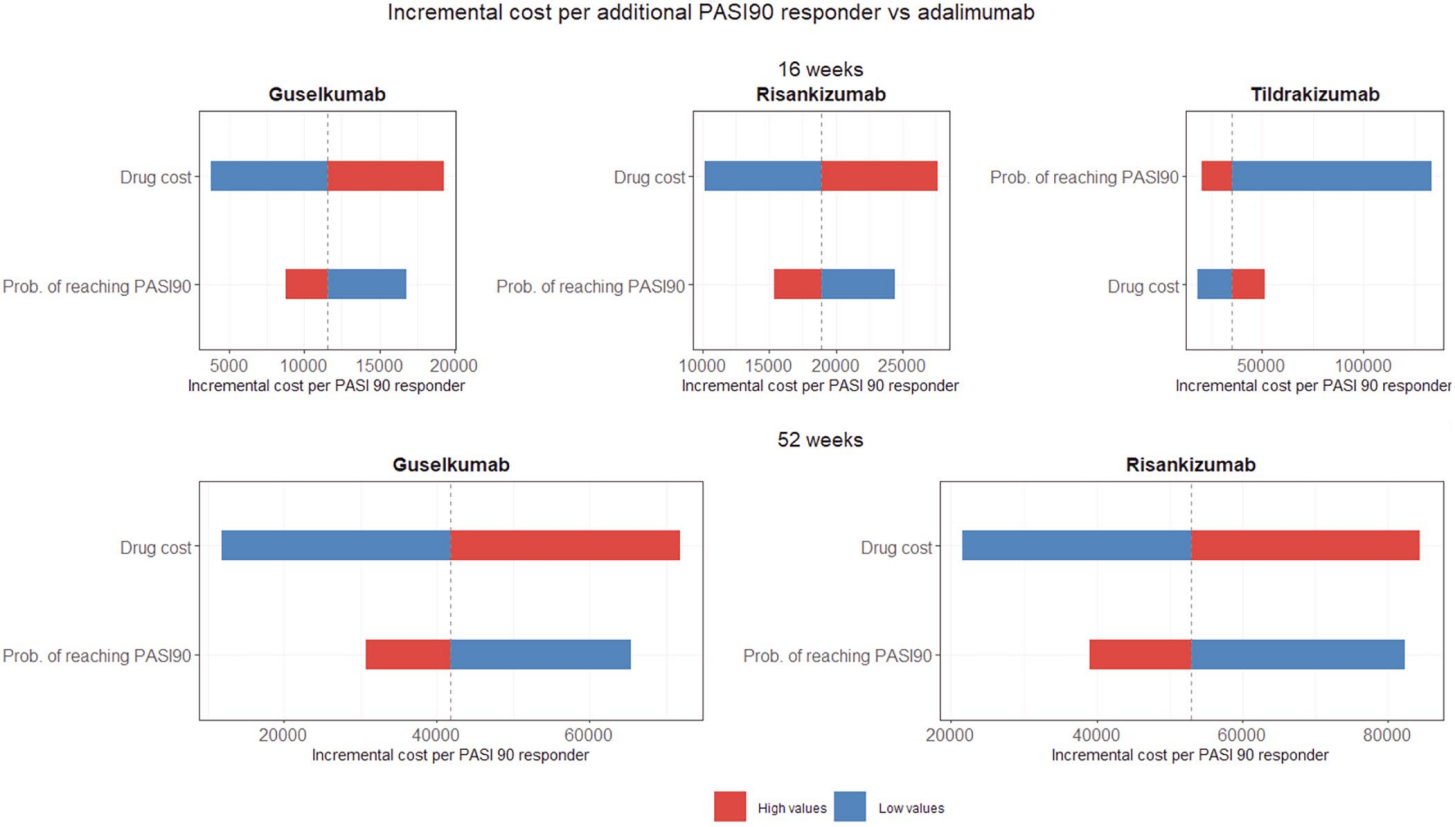
Tornado diagram showing the variation in incremental cost per PASI90 responder with variation in the value of parameters. All treatments are compared against adalimumab as the reference comparator.

Usually, it is useful to convert effectiveness into a monetary unit and then compare the net monetary benefit (NMB) of each biological treatment across a range of thresholds, which serve as cut‐offs for determining what is considered cost‐effective (€35,061.50). In Figure 3, each point represents a pair of incremental cost and incremental effectiveness with uncertainty around costs and effectiveness accounted for in the model (cost variation percentages: 30%, correlation coefficient: 0.4). The points illustrate whether the incremental effectiveness, when converted into monetary terms, exceeds the incremental cost at a fixed threshold incremental cost per PASI90 responder of €20,000, representing the willingness‐to‐pay (WTP) per unit of incremental effectiveness.

**FIGURE 3:**
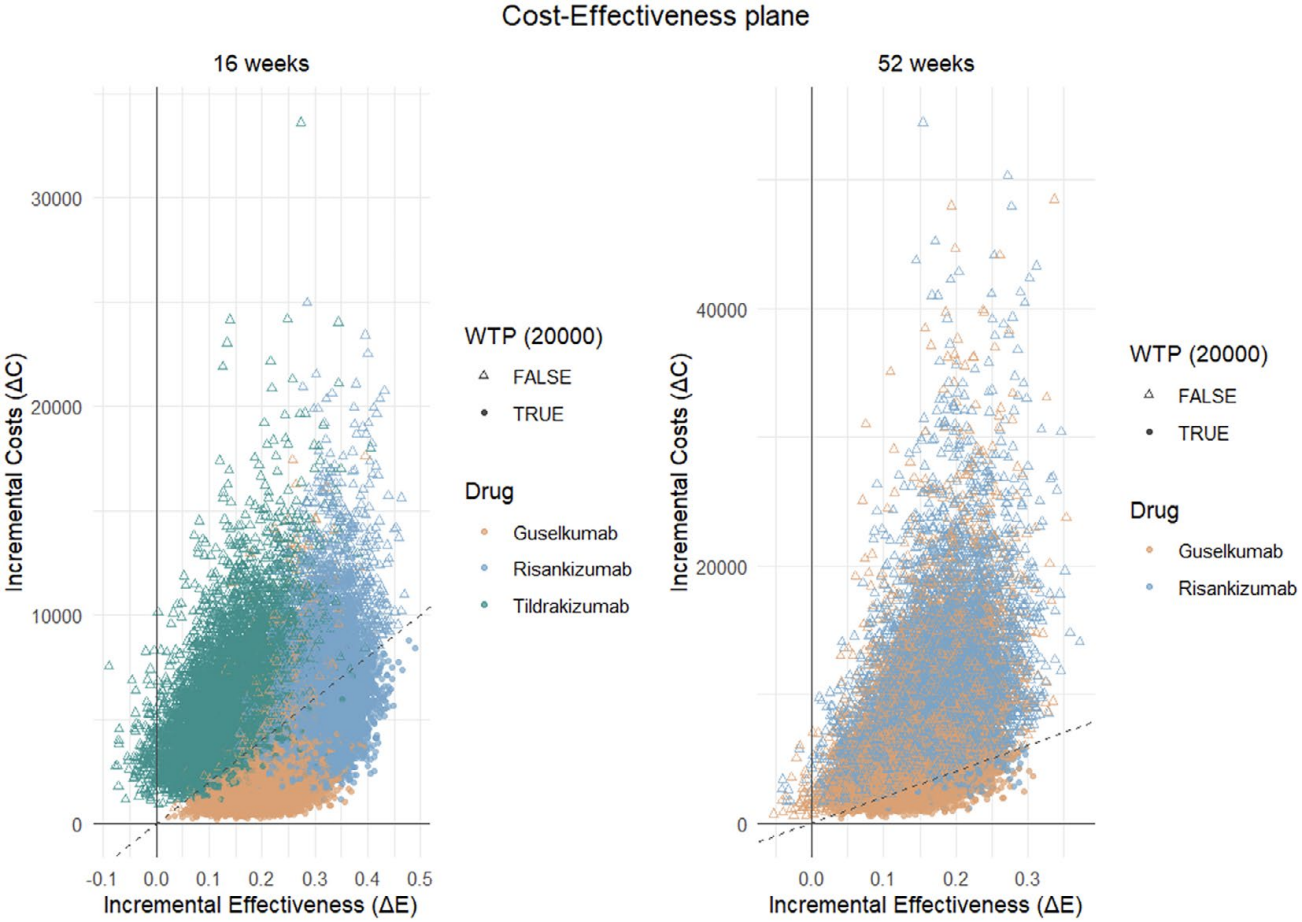
Cost‐effectiveness scatter plot showing simulated pairs of incremental cost and incremental effectiveness for each treatment, generated through Probabilistic Sensitivity Analysis (PSA) with a 30% cost variation and a correlation coefficient of 0.4. All treatments are compared against adalimumab as the reference comparator.

Pairs falling below the threshold dotted line are shown as solid dots, while those above the line are represented by empty triangles. At 16 weeks, guselkumab has 90.2% of 10,000 samples falling below the threshold line, followed by 64.1% for risankizumab and 13.3% for tildrakizumab. In contrast, at 52 weeks, only 15.9% of guselkumab samples and 4.4% of risankizumab samples lie below the threshold line. This decrease reflects the higher incremental cost per PASI90 responder point estimates observed at 52 weeks, driven by both an increased incremental cost and a reduced incremental effectiveness for these biological treatments. Cost‐effectiveness acceptability curves (CEACs) display the proportion of cases in which each biological treatment has the highest NMB at each threshold we considered during the analyses. At 16 weeks, the CEACs indicate that guselkumab has the highest probability of being cost‐effective compared to other biologic treatments, up to an incremental cost per PASI90 responder threshold of €36,400 (Figure 4). Beyond this threshold, risankizumab becomes the treatment with the highest probability of being cost‐effective. This switch is a direct consequence of the greater clinical benefit associated with risankizumab, as reflected by its lower NNT, which is the closest to one between the three biological treatments. At 52 weeks, for every threshold considered (0–€50,000), guselkumab consistently shows a higher probability of being cost‐effective compared to risankizumab.

**FIGURE 4:**
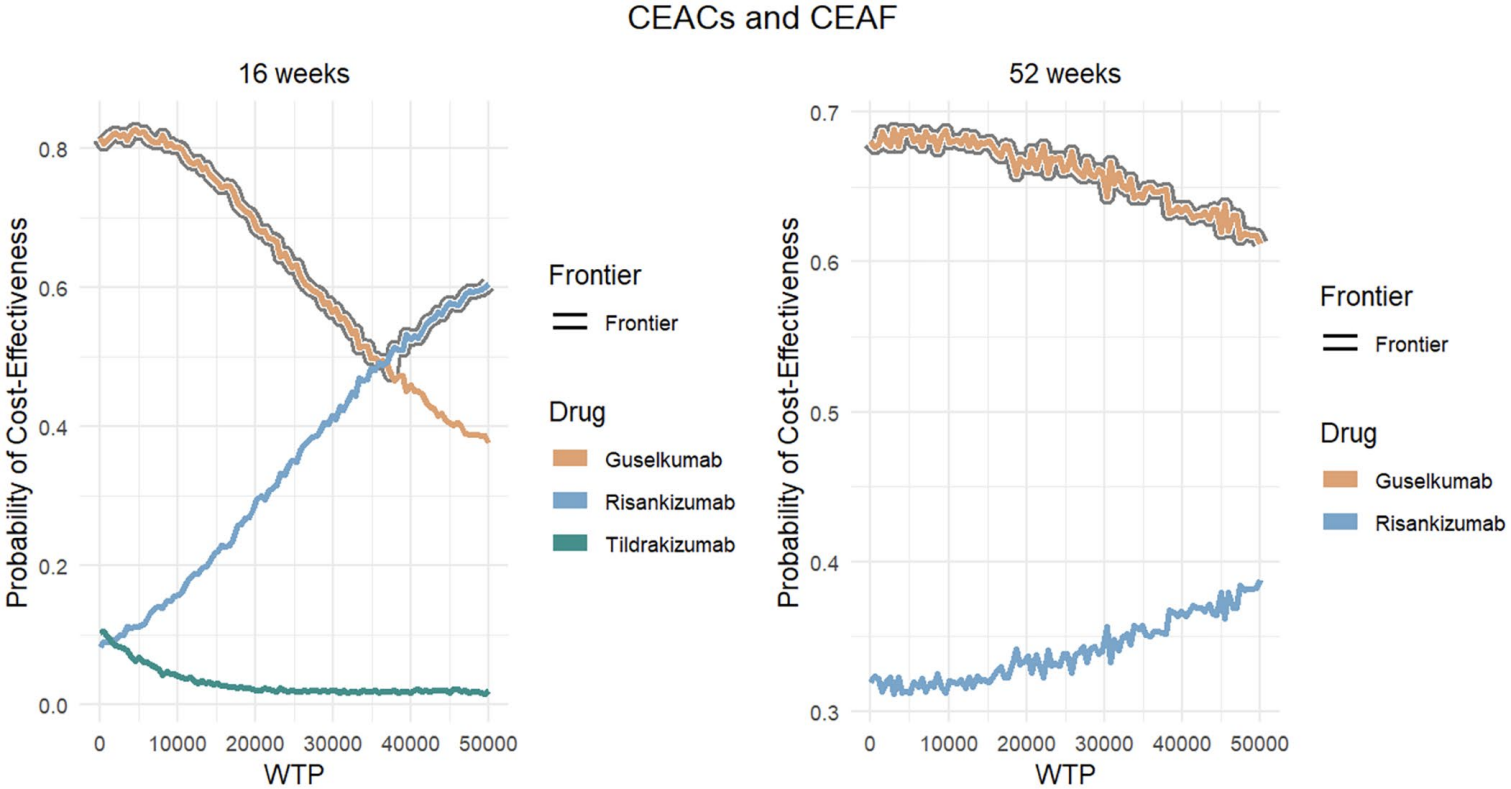
Cost‐effectiveness acceptability curves (CEACs) and cost‐effectiveness acceptability frontier (CEAF) for the treatments under analysis at 16 and 52 weeks. All treatments are compared against adalimumab as the reference comparator.

To determine which treatment offers the highest expected net monetary benefit, the cost‐effectiveness acceptability frontier (CEAF) is typically used [14]. The CEAF, depicted by the gray highlighted frontier line in the figures, illustrates the optimal treatment option for each considered WTP threshold. At 16 weeks, guselkumab tended to be more cost‐effective than the other IL‐23 inhibitors up to a WTP of €37,400, after which risankizumab becomes the preferred treatment. At 52 weeks, guselkumab remained the option with a relatively lower cost per responder compared with the other IL‐23 inhibitors across all WTP values.

## 4 Discussion

The effectiveness of anti IL‐23 for treatment of moderate to severe psoriasis has been widely reported in randomized clinical trials and real‐world studies, including patients with multiple comorbidities [15, 16, 17, 18, 19]. In the present study, we observed high PASI90 response rates and long‐term maintenance, with 82.4% of patients treated with guselkumab, 84.1% with risankizumab, and 77.6% with tildrakizumab achieving PASI90 at Week 52.

In addition, our study demonstrated that the IL‐23 inhibitors had the lowest NNT values to reach PASI90, with clinical benefit sustained over a 52‐week treatment, compared to adalimumab biosimilars at both 16 and 52 weeks. Among the IL‐23 class, the lowest NNT to achieve PASI90 (highest effectiveness) was observed with risankizumab at Week 16 (NNT = 3.34, 95% CI = 2.50–5.04), and confirmed in the long‐term at Week 52 (NNT = 6.00; 95% CI = 3.62–17.49).

A recent meta‐analysis showed that IL‐17 inhibitors (secukinumab, brodalumab, and ixekizumab) have the lowest NNT values, exhibiting the highest efficacy in achieving PASI responses at early time points. However, after 48–52 weeks of continuous treatment, they did not significantly differ from IL‐23 inhibitors such as risankizumab and guselkumab. Therefore, IL‐17 inhibitors may be particularly effective for achieving rapid clinical response, while IL‐23 inhibitors maintain comparable efficacy over the long term [20].

Biological drugs have a considerable impact on healthcare expenditure. In this context, the need to contain healthcare costs has led many health authorities to require the use of lower‐cost treatments, such as adalimumab biosimilars, as first‐line biologic therapy for moderate‐to‐severe psoriasis [21].

Our cost per responder analysis showed that IL‐23 inhibitors and particularly risankizumab have the highest average cost at both Week 16 and 52, but also the greatest incremental effectiveness compared to adalimumab biosimilars. Guselkumab demonstrated the most favorable incremental cost per PASI90 responder at both time points, while risankizumab showed superior incremental effectiveness. At 16 weeks, guselkumab was shown to be superior to tildrakizumab, as it was both more effective and less costly in incremental terms. Probabilistic sensitivity analysis confirmed that guselkumab had the highest probability of being cost‐effective up to a WTP threshold of approximately €37,000, beyond which risankizumab became the preferred option due to its greater clinical benefit. At Week 52, guselkumab consistently remained the option with a relatively lower cost per responder compared with the other IL‐23 inhibitors across all WTP values. These findings were supported by previous economic evaluations showing that the high clinical benefit of risankizumab, despite its higher drug cost, remained cost‐effective compared to adalimumab [22]. Similarly, another study demonstrated that, among several biologics, guselkumab offered the lowest cost per PASI90 responder at Week 16, reinforcing its favorable short‐term cost‐effectiveness [23].

This study has some limitations, including its retrospective design and restriction to only two hospitals, which may limit generalizability. Moreover, in routine practice in Italy, adalimumab biosimilars are often selected as first‐line biologic therapy due to cost and reimbursement considerations, resulting in a substantially higher proportion of biologic‐naive patients than in the IL‐23 inhibitor groups, where prior biologic exposure was more common and could have affected response. Moreover, the number of evaluable patients at Week 52 differed across groups. PASI data at Week 52 were available for 83/165 adalimumab‐treated patients, 193/200 guselkumab‐treated patients, 145/176 risankizumab‐treated patients, and 58/75 tildrakizumab‐treated patients. The reduced denominators at Week 52 reflect routine clinical practice attrition due to missing follow‐up assessments and/or treatment discontinuation; since outcomes were analyzed as observed without imputation, Week 52 PASI90 rates and derived NNTs should be interpreted cautiously. Moreover, the unequal sample sizes across treatment groups may have introduced selection bias and affected comparability. In addition, the cost‐per‐responder analysis only considered drug acquisition costs; other direct and indirect costs, although likely to be small in this patient population, were not included and could affect total cost estimates. Another limitation of this study is that results are restricted to patients with continuous treatment persistence up to the analyzed time points; therefore, cost estimates may not fully reflect scenarios characterized by treatment discontinuation or non‐persistence.

However, this study offers an original and relevant contribution by comparing IL‐23 inhibitors with adalimumab biosimilars since it integrates efficacy data from pivotal clinical trials and real‐world studies. Our large sample size enhances the robustness and generalizability of the findings, providing a comprehensive and clinically meaningful perspective. Cost‐effectiveness modeling, including one‐way deterministic sensitivity analysis and probabilistic sensitivity analysis, offers solid support for evidence‐based healthcare decisions. The study stands out for the originality of its comparison and the direct applicability of its findings to real‐world settings.

In conclusion, our real‐world data confirm the clinical superiority of IL‐23 inhibitors over anti‐TNF biosimilars for the treatment of moderate‐to‐severe psoriasis, with risankizumab showing the highest efficacy and guselkumab emerging as the option associated with the highest probability of being cost‐effective.

## Conflicts of Interest

Ketty Peris has served on an advisory board, received honoraria for lectures, and/or research grants for AbbVie, Almirall, Lilly, Galderma, Leo Pharma, Pierre Fabre, Novartis, Sanofi, Sun Pharma, and Janssen. The other authors have no conflicts of interest to declare. Clara De Simone has received consulting fees, honoraria, and support for attending meetings from AbbVie, Lilly, Janssen, UCB, Novartis, Leo Pharma, Sanofi, and Almirall. Giacomo Caldarola has received consulting fees, honoraria, and support for attending meetings from AbbVie, Lilly, Almirall, Janssen, UCB, Novartis, and Leo Pharma. Niccolò Gori served as advisory board member and received honoraria for lectures for AbbVie, Sanofi, Leo‐Pharma, Almirall, Lilly, Pfizer, Novartis and Galderma. Marco Galluzzo has acted as a speaker and/or consultant for AbbVie, Almirall, Eli‐Lilly, Johnson & Johnson, LeoPharma, Novartis, Pfizer and Sanofi, outside the submitted work. Luca Bianchi declares to have acted as a speaker and/or consultant for AbbVie, Almirall, Eli‐Lilly, Johnson & Johnson, LeoPharma, Novartis, Pfizer, Sanofi and UCB outside the submitted work. Marina Talamonti has acted as a speaker and/or consultant for AbbVie, Almirall, Eli‐Lilly, Johnson & Johnson, LeoPharma, Novartis, and Sanofi, outside the submitted work. Other authors have no relevant conflicts of interest to declare.

## Supporting information


**Figure S1:** Cost‐Effectiveness Acceptability Curves (CEACs) for the evaluated treatments at 16 weeks, considering alternative assumptions of cost uncertainty (cost standard deviation: 0.2, 0.3, 0.4) and correlation between incremental cost and effectiveness (*ρ* = −0.1, 0.1, 0.3, 0.5). All treatments are compared against adalimumab as the reference comparator.
**Figure S2:** Cost‐Effectiveness Acceptability Curves (CEACs) for the evaluated treatments at 52 weeks, considering alternative assumptions of cost uncertainty (cost standard deviation: 0.2, 0.3, 0.4) and correlation between incremental cost and effectiveness (*ρ* = −0.1, 0.1, 0.3, 0.5). All treatments are compared against adalimumab as the reference comparator.
**Figure S3:** Cost‐Effectiveness Acceptability Curves (CEACs) and Cost‐Effectiveness Acceptability Frontier (CEAF) for the treatments under analysis at 16 weeks and 52 weeks.
**Table S1:** Cost of drugs and dosing schedules over 16 weeks of treatment.
**Table S2:** Patients' clinical characteristics and response rates at week 16 and 52 of treatment.

## Data Availability

The data that support the findings of this study are available from the corresponding author upon reasonable request.
