# Supplementary material for: Superior Clinical and Economic Value of IL‐23 Inhibitors Versus Adalimumab Biosimilars in Plaque Psoriasis
Source: Int J Dermatol. 2026 Mar 29;65(11):2362–70. doi: 10.1111/ijd.70371 (PMC13646478; doi:10.1111/ijd.70371)
Supplement: Supplementary file 1 [file IJD-65-2362-s001.docx]

| **Treatment** | **Package** | **Price** | | **Cost per mg** | **Treatment schedule** | **N. of administrations W16** |
| --- | --- | --- | --- | --- | --- | --- |
|  |  | **Gross ex-factory** | **Net ex-factory** |  |  |  |
| WAP Adalimumab biosimilars 40 mg | 1 syringe 40 mg | / | €309.03 | €7.73 | Initial dose of 80 mg s.c. followed by 40 mg after 7 days, then 40 mg every 2 weeks | 10 |
| Risankizumab 150 mg | 1 syringe 150 mg | €3,230.00 | €2,915.08 | €19.43 | Initial dose 150 mg s.c, followed by 150 mg after 4 weeks, then 150 mg every 12 weeks | 3 |
| Tildrakizumab 100 mg | 1 syringe 100 mg | €3,118.45 | €2,814.40 | €28.14 | Initial dose of 100 mg s.c, followed by 100 mg after 4 weeks, then 100 mg every 12 weeks. | 3 |
| Guselkumab 100 mg | 1 syringe 100 mg | €2,062.98 | €1,861.84 | €18.62 | Initial dose of 100 mg s.c, followed by 100 mg after 4 weeks, then 100 mg every 8 weeks | 3 |

**Table S1** – Cost of drugs and dosing schedules over 16 weeks of treatment.

|  | Adalimumab Biosimilar | Risankizumab | Tildrakizumab | Guselkumab |
| --- | --- | --- | --- | --- |
| Pharmaceutical form | 1 syringe of 40 mg | 1 syringe of 150 mg | 1 syringe of 100 mg | 1 syringe of 100 mg |
| Gross ex-factory price | — | €3,230.00 | €3,118.45 | €2,062.98 |
| Net ex-factory price | €309.03 | €2,915.08 | €2,814.40 | €1,861.84 |
| Cost per mg | €7.73 | €19.43 | €28.14 | €18.62 |
| Dosing schedule | Initial dose of 80 mg s.c. followed by 40 mg after 7 days, then 40 mg every 2 weeks | Initial dose 150 mg s.c, followed by 150 mg after 4 weeks, then 150 mg every 12 weeks | Initial dose of 100 mg s.c, followed by 100 mg after 4 weeks, then 100 mg every 12 weeks. | Initial dose of 100 mg s.c, followed by 100 mg after 4 weeks, then 100 mg every 8 weeks |
| Number of administrations (W16) | 10 | 3 | 3 | 3 |
| Total dose (mg) | 400 mg | 450 mg | 300 mg | 300 mg |
| Total cost (W16) | €3,090.30 | €8,745.24 | €8,443.20 | €5,585.52 |

**s.c.= subcutaneous**

**Table S2.** Patients’ clinical characteristics and response rates at week 16 and 52 of treatment

|  | Total  n=616 | Adalimumab  165 | Guselkumab  200 | Tildrakizumab  75 | Risankizumab  176 |
| --- | --- | --- | --- | --- | --- |
| Age [mean (±SD)] | | | | | |
| Female [n(%)] | 310 (50,3) | 83 (50,3) | 98 (49) | 40 (53.3) | 89 (50.6) |
| Male [n(%)] | 306 (49,7) | 82 (49,7) | 102 (51) | 35 (46.7) | 87 (49.4) |
| Family history of PsO [n(%)] | 257 (41.7) | 69 (41,8) | 99 (49,5) | 28(37.3) | 61 (34.6) |
| **Naive to systemic therapy** |  |  |  |  |  |
| Yes [n (%)] | 352 (57.1) | 159 (96,4) | 75 (37,5) | 52 (69.3) | 66 (37.5) |
| one previous biological therapy [n (%)] | 156 (25,3) | 5 (3,03) | 63 (31,5) | 21 (28.0) | 67 (38.1) |
| more than one previous biological therapy [n (%)] | 108 (17.5) | 1 (0,6) | 62 (31) | 2 (2.7) | 43 (24.4) |
|  | | | | | |
| Mean PASI at week 0 [mean (±SD)] | 13.7 (9.2) | 11.8 (7.2) | 14.5 (11.1) | 12.4 (6.7) | 15.1 (9.1) |
| Mean PASI score week 16 [mean (±SD)] | 2.3 (3.4) | 3.4 (3.8) | 2.2 (3.3) | 2 (2.6) | 1.4 (3.0) |
| Mean PASI score week 52 [mean (±SD)] | 0.9 (2.12) | 1.7 (3.2) | 0.7 (1.7) | 1.1 (2.2) | 0.6 (1.7) |
| PASI 90 response at week 16 [n (%)] | 350 (56.8) | 65 (39.4) | 122 (61) | 41 (54.7) | 122 (69.3) |
| PASI 90 response at week 52 [n (%)] | 382 (62) | 56 (67.5) | 159 (82.4) | 45 (77.6) | 122 (84.1) |
| PASI 90 response at week 104 [n (%)] | 259 (42) | 23 (85.2) | 121 (84.6) | 33(89.2) | 82 (85.4) |
| **Drug Discontinuation at week 52** [n (%)] | 88 (14.3) | 55 (33.3) | 7 (3.5) | 10 (13.3) | 16 (9.1) |
| Inefficacy [n (%)] | 65(10.6) | 48 (24) | 3 (1.5) | 6 (8.0) | 8 (4.5) |
| Stable efficacy [n (%)] | 1 (0.2) | 0 | 0 | 0 (0) | 1 (0.6) |
| Adverse event [n (%)] | 6 (1) | 4 (2) | 1 (0.5) | 1 (1.3) | 0 (0) |
| Lost to follow up [n (%)] | 16 (2.6) | 3 (1.5) | 3 (1.5) | 3 (4.0) | 7 (4.0) |

Ref.: Data are relative to adalimumab

RR: relative risk

ARR: absolute risk reduction

NNT: number needed to treat (1/ARR)

**Figure S1.** Cost-Effectiveness Acceptability Curves (CEACs) for the evaluated treatments at 16 weeks, considering alternative assumptions of cost uncertainty (cost standard deviation: 0.2, 0.3, 0.4) and correlation between incremental cost and effectiveness (ρ = –0.1, 0.1, 0.3, 0.5). All treatments are compared against adalimumab as the reference comparator.

**Figure S2.**  Cost-Effectiveness Acceptability Curves (CEACs) for the evaluated treatments at 52 weeks, considering alternative assumptions of cost uncertainty (cost standard deviation: 0.2, 0.3, 0.4) and correlation between incremental cost and effectiveness (ρ = –0.1, 0.1, 0.3, 0.5). All treatments are compared against adalimumab as the reference comparator.

A new PSA was conducted using 10,000 samples, assuming a beta distribution for effectiveness. A log-normal distribution was assumed for costs with the mean set to the average cost and a standard deviation corresponding to a 30% variation in cost of each treatment. As shown in figure S3, at 16 weeks, the CEACs indicate that adalimumab has the highest probability of being cost-effective compared to other treatments up to threshold value of €10,500. Beyond that threshold, guselkumab has highest probability of being cost-effective within a range from a WTP of €10,500 to €35,500. Finally, beyond €35,500, risankizumab becomes the treatment with the highest probability of being cost-effective. At 52 weeks, adalimumab shows a higher probability of being cost-effective compared to biological treatments up to WTP of €37,900. At 16 weeks, guselkumab is the most cost-effective option within a range from a WTP of €12,000 to €37,400, after which risankizumab becomes the preferred treatment. At 52 weeks, adalimumab remains the most cost-effective option up to a WTP of €41,400.

**Figure S3.** Cost-Effectiveness Acceptability Curves (CEACs) and Cost-Effectiveness Acceptability Frontier (CEAF) for the treatments under analysis at 16 weeks and 52 weeks.
